# Identification, Expression, and Functional Analysis of the Fructokinase Gene Family in Cassava

**DOI:** 10.3390/ijms18112398

**Published:** 2017-11-12

**Authors:** Yuan Yao, Meng-Ting Geng, Xiao-Hui Wu, Chong Sun, Yun-Lin Wang, Xia Chen, Lu Shang, Xiao-Hua Lu, Zhan Li, Rui-Mei Li, Shao-Ping Fu, Rui-Jun Duan, Jiao Liu, Xin-Wen Hu, Jian-Chun Guo

**Affiliations:** 1 Key Laboratory of Biology and Genetic Resources of Tropical Crops, Ministry of Agriculture, Institute of Tropical Bioscience and Biotechnology, Chinese Academy of Tropical Agricultural Sciences, Haikou 571101, China; yaoyuan@itbb.org.cn (Y.Y.); liruimei@itbb.org.cn (R.-M.L.); fushaoping@itbb.org.cn (S.-P.F.); duanruijun@itbb.org.cn (R.-J.D.); liujiao@itbb.org.cn (J.L.); 2 College of Agriculture, Hainan University, Haikou 570228, China; mengtinggeng8908@gmail.com (M.-T.G.); wyl349399603@gmail.com (Y.-L.W.); cx15298909742@gmail.com (X.C.); shanglu0603@gmail.com (L.S.); yimihua2514@gmail.com (X.-H.L.); lizhan71707@gmail.com (Z.L.); 3 Prisys Biotechnologies Company Limited, Shanghai 201203, China; xiaohui.wu@prisysbiotech.com; 4 College of Life Science and Biotechnology, Heilongjiang Bayi Agricultural University, Daqing 163319, China; s780347812@gmail.com

**Keywords:** cassava, fructokinase, gene expression, yeast complementation, enzyme activities

## Abstract

Fructokinase (FRK) proteins play important roles in catalyzing fructose phosphorylation and participate in the carbohydrate metabolism of storage organs in plants. To investigate the roles of FRKs in cassava tuber root development, seven *FRK* genes (*MeFRK1*–*7*) were identified, and *MeFRK1*–*6* were isolated. Phylogenetic analysis revealed that the *MeFRK* family genes can be divided into α (*MeFRK* *1*, *2*, *6*, *7*) and β (*MeFRK* *3*, *4*, *5*) groups. All the MeFRK proteins have typical conserved regions and substrate binding residues similar to those of the FRKs. The overall predicted three-dimensional structures of MeFRK1–6 were similar, folding into a catalytic domain and a β-sheet ‘‘lid” region, forming a substrate binding cleft, which contains many residues involved in the binding to fructose. The gene and the predicted three-dimensional structures of *MeFRK3* and *MeFRK4* were the most similar. *MeFRK1–6* displayed different expression patterns across different tissues, including leaves, stems, tuber roots, flowers, and fruits. In tuber roots, the expressions of *MeFRK3* and *MeFRK4* were much higher compared to those of the other genes. Notably, the expression of *MeFRK3* and *MeFRK4* as well as the enzymatic activity of FRK were higher at the initial and early expanding tuber stages and were lower at the later expanding and mature tuber stages. The FRK activity of MeFRK3 and MeFRK4 was identified by the functional complementation of triple mutant yeast cells that were unable to phosphorylate either glucose or fructose. The gene expression and enzymatic activity of *MeFRK3* and *MeFRK4* suggest that they might be the main enzymes in fructose phosphorylation for regulating the formation of tuber roots and starch accumulation at the tuber root initial and expanding stages.

## 1. Introduction

In higher plants, sucrose is the end product of photosynthesis and is the main carbohydrate transport form in phloem [1,2]. Sucrose metabolism plays a central role in plant growth and development, yield formation, and response to biotic and abiotic stresses [1,3]. Invertase and sucrose synthase are the two key enzymes that catalyze the breakdown of sucrose into hexose [4]. Invertase irreversibly cleaves sucrose into fructose and glucose [5]. Sucrose synthase reversibly converts sucrose and uridine diphosphate (UDP) into fructose and uridine diphosphate glucose (UDPG) [6]. Fructose accounts for half of the total products of sucrose degradation, and in addition to being a carbon and energy source, it has been recognized as a key signaling molecule in plants [7,8].

Fructose must first be phosphorylated to fructose-6-phosphate by fructokinases (FRKs) or hexokinases (HXKs) before undergoing further metabolism [9]. The affinity of FRKs for fructose is much higher than HXKs [10,11], which suggests that fructose in plants might be mainly phosphorylated by FRKs. Plant FRKs belong to the pfkB family of carbohydrate kinases, containing a di-gly (GG) motif at the N-terminal region and GAGD motif at the C-terminal region [12]. Two to eight *FRK* genes exist in different plant species. Fructokinase genes have been cloned from many species, such as *Solanum lycopersicon* [13], *Oryza sativa* [14], *Citrus reticulata* [15], *Arabidopsis thaliana* [12], *Zea mays* [16], and *Populus tremula* [17].

The functions of plant FRKs have been characterized in some species, including their roles in plant growth and development. In tomato, *FRK1*-antisense plants exhibited delayed flowering at the first inflorescence; *FRK2*-antisense plants resulted in inhibition of stem and root growth, reduction in flower and fruit numbers, and reduction in seed numbers per fruit [18]; *FRK3*-antisense in plants had no effect on plant growth, but mildly affected the xylem development, reduced the hydraulic conductivity, and caused the abortion of flowers [13]; *FRK4* was specifically activated in pollen grains throughout the later stages of anther development and during pollen germination [19]. FRKs might have an important role in plant responses to abiotic stress. Two rice *FRK* genes had different expression patterns under aerobic and anaerobic conditions: *OsFK1* was mainly expressed under aerobic conditions, whereas *OsFK2* was induced under anaerobic conditions [20]. In maize, the expression of *FRK2* was found to increase under short-term salt stress conditions [21]. It has been reported that the product of FRK is catalyzed by cytosolic phosphoglucose isomerase (PGI) to form glucose 6-phosphate, which can be transferred to the amyloid by hexose phosphate translocator. Glucose 6-phosphate is catalyzed by plastidial phosphoglucose mutase (PGM) to form glucose 1-phosphate, and this product is converted to adenosine diphosphate glucose (ADPglucose) catalyzed by ADPglucose pyrophosphorylase (AGPase) [22]. ADPglucose is the substrate for starch synthesis [23]. Therefore, fructokinase may play a role in starch accumulation in the sink organs of crops. Plant FRKs are found in two locations: plastids and the cytoplasm [9,11]. The existence of plastidic FRK may be important for starch synthesis, since starch is accumulated in plastids. Fructokinase is involved in the early stage of wheat grain development [24]. During maize seed development, *ZmFrk1* is highly expressed 15 days after pollination, when starch quickly accumulates in seeds, indicating that FRK plays a role in starch synthesis in the endosperm [16]. The activity of the FRK protein in *S. tuberosum* increases in association with tuberization [25]. Antisense inhibition of the FRK *StFK1* gene in *S. tuberosum* resulted in a reduced rate of tuberization and total tuber yield [22]. These results indicate that FRK might be involved in the tuberization and starch accumulation in plant tubers.

Cassava (*Manihot esculenta* Crantz) is a tuber root crop with a 70–90% dry weight of starch in the tuber root, and serves as a dietary staple food for more than 700 million people worldwide [26,27]. Photosynthetic carbon assimilation in cassava is extremely high, at 43 µmol CO_2_/m^2^/s, which is twice the photosynthesis rate in rice, of around 20 µmol CO_2_/m^2^/s. However, the yield of cassava is far from its maximum yield potential [28]. It has been reported that enhanced ADP-glucose pyrophosphorylase (AGPase) activity in cassava tuber root, using transgenic technology, can increase its tuber root number and dry weight [28]. This result suggests that an increase in the carbohydrate metabolism-related enzyme activity in tuber root could contribute to tuber root growth and starch accumulation. Recently, proteomics profiling revealed increased levels of FRKs as well as starch and sucrose metabolism proteins in tuber root during cassava root tuberization [29]. However, the regulation of fructose phosphorylation in cassava has not yet been characterized. In this study, six cDNAs of FRK were isolated from cassava. The evolutionary relationships, exon-intron structure, chromosome distribution, and protein three-dimensional (3D) structure of all six genes were investigated. To elucidate the putative roles of *FRK* genes in cassava, their gene expression patterns and enzyme activities during cassava tuber root development were investigated. Finally, the FRK activity of MeFRK3 and MeFRK4 were investigated through a functional complementation experiment in the triple mutant yeast cells, YSH74-3C (*hxk1*, *hxk2*, *glk1*), which are unable to phosphorylate either glucose or fructose. These results can shed light on the putative roles of FRK in sucrose metabolism in cassava tuber root.

## 2. Results

### 2.1. Cloning and Sequence Analysis of the Fructokinase Gene Family from Cassava

**Figure 1:**
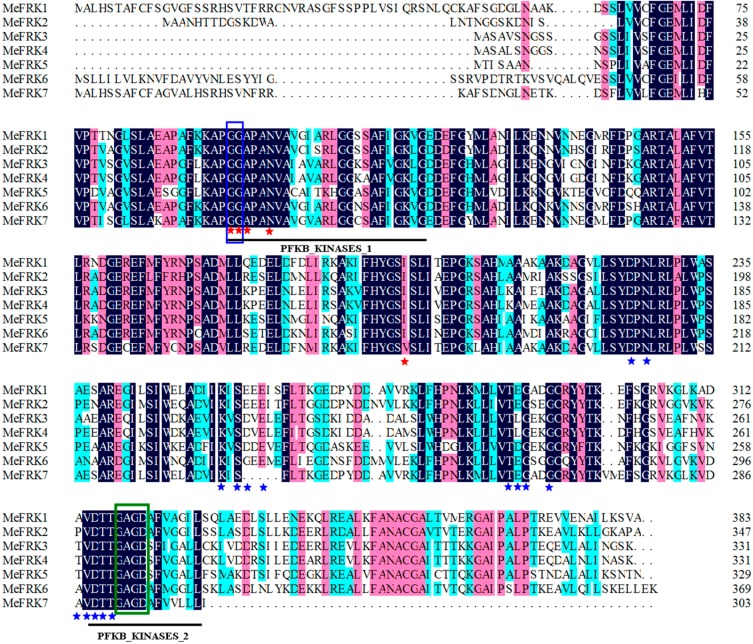
Alignment of the deduced amino acid sequences of the six cassava fructokinases (FRKs). Two conserved domains (pfkb1 and pfkb2) of FRKs are indicated by black lines. The di-gly (GG) and G/AXGD motifs are indicated by blue and green boxes, respectively. Proposed key substrate binding residues of fructose and ATP are indicated by red and blue asterisks, respectively. Dark-blue shading, pinkish shading and light blue shading reflect 100%, 75% and 50% amino acid residues conservation, respectively.

Seven *FRK* genes were initially identified in the cassava genome database (*MeFRK1*–*7*). Full-length coding sequences for six *MeFRKs* (*MeFRK1*–*6*) were cloned from cassava cultivar SC8 by reverse transcription polymerase chain reaction (RT-PCR), using a cDNA template mixture of leaf, flower, and tuber root. An endogenous transcript for the *MeFRK7* gene in the various samples was not detectable, indicating that the gene is inactive in the examined tissues. The cDNA and the deduced amino acid sequences of the six *MeFRKs* described in this study were deposited in GenBank under the following accession numbers: *MeFRK1* (AJK93565), *MeFRK2* (AJK93566), *MeFRK3* (AJK93567), *MeFRK4* (MeFRK4), *MeFRK5* (KR338981), and *MeFRK6* (AJK93569). The deduced amino acids of MeFRKs are between 383 and 303 a.a, and their theoretical pIs range from 6.62 to 4.99 (Table 1). The molecular mass of MeFRK1 is the largest (41.2 kDa), whereas the smallest one is MeFRK7 (32.9 kDa). Alignment analysis of the amino acids showed that MeFRKs share 46.69–93.05% identity among all family genes. Interestingly, MeFRK3 and MeFRK4 share the highest identity at 93.05% and have the same deduced amino acid length and similar molecular mass. MeFRK2, MeFRK5, and MeFRK6 were predicted to be located in the cytoplasmic, whereas MeFRK1, MeFRK3, MeFRK4, and MeFRK7 were predicted to be located in the plastid. All MeFRKs have two conserved domains of FRK, i.e., pfkB1 and pfkB2, which suggests that these proteins are members of the FRK family. The di-gly (GG) motif is in the N-terminal region, and the GAGD motif is in the C-terminal region. These conserved motifs are important for the enzyme activity of FRK. MeFRKs contain multiple conserved residues, which might play a key role in binding fructose and ATP (Figure 1).

### 2.2. Structure Analysis and Chromosomal Distribution of the MeFRK Family Genes

**Figure 2:**
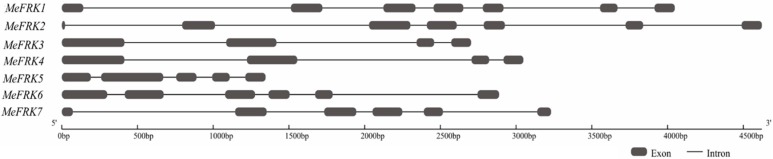
Exon-intron structure of the seven fructokinases in cassava. Introns are shown as black lines, and exons are shown as boxes.

**Figure 3:**
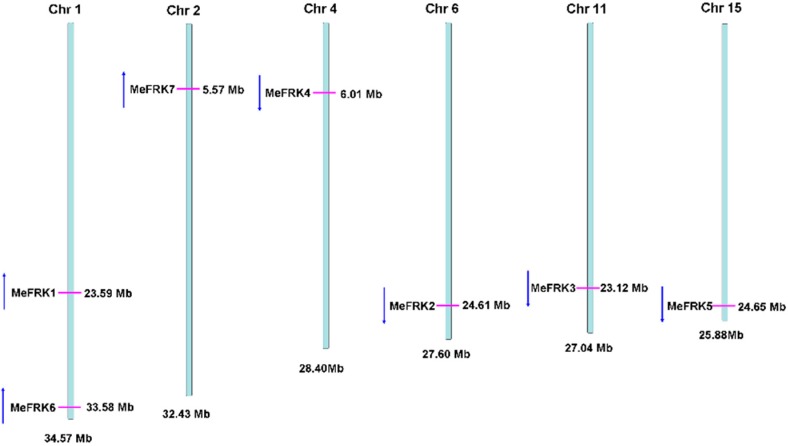
Genome-wide distribution and orientation of the *MeFRK* genes on cassava chromosomes. Chromosome numbers are indicated at the top of each bar. The red lines on the cassava chromosomes indicate the positions of the *MeFRK* genes. The blue arrows indicate the gene orientation.

The gene structures of the *MeFRK* genes were studied by aligning the genomic sequence from the cassava genome database (Available online: http://www.phytozome.net/cassava) and the CDS region for each *MeFRK* gene. The number of exons in *MeFRK* genes ranges from four to seven (Figure 2). *MeFRK1* and *MeFRK2* have seven exons, and the first exon of *MeFRK2* is the smallest one (22 bp) among all the exons in *MeFRKs*. *MeFRK3* and *MeFRK4* share similar gene structures: both contain four exons, and the length of each exon is the same. The gene length of *MeFRK5* is the smallest among all the *MeFRKs*, with five exons. *MeFRK6* and *MeFRK7* have six exons. The chromosomal distribution and orientation of *MeFRK* genes were obtained by identifying their chromosomal position, as per version 6.1 of the cassava genome database. The seven *MeFRK* genes were mapped to six chromosomes of the cassava genome (Figure 3). No tandem duplication of the cassava FRK genes was found. *MeFRK1* and *MeFRK6* were mapped in Chromosome 1, with opposite orientation to the other *MeFRK* genes. *MeFRK7* was mapped in Chromosome 2. *MeFRK2*–*5* have the same orientation and are present in Chromosomes 4, 6, 11, and 15, respectively. Interestingly, most of the *MeFRK* genes are distributed at the end of chromosomes, such as *MeFRK2*–*7*.

### 2.3. Phylogenetic Analysis of MeFRK Genes

**Figure 4:**
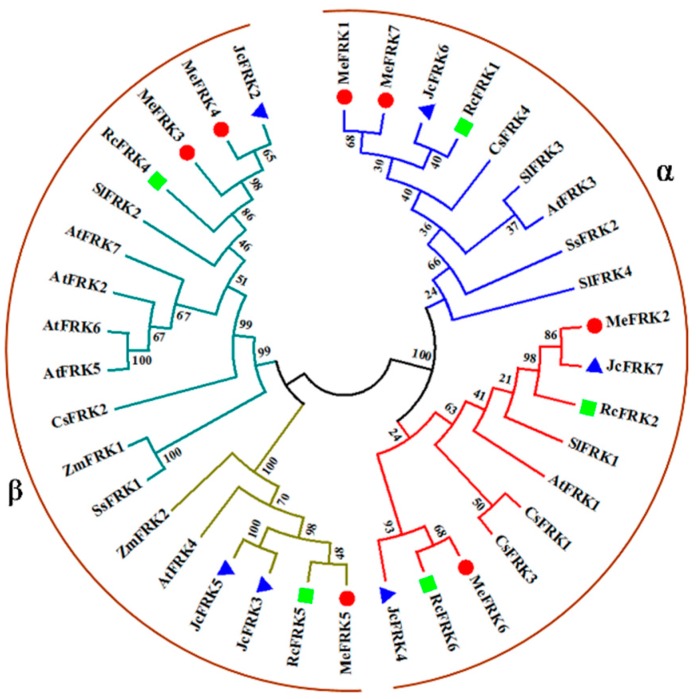
Phylogenetic analysis of the FRK proteins from *M. esculenta*, *J. curcas*, *R. communis, S. lycopersicum*, *A. thaliana*, *C. Sinensis*, *S. spontaneum*, and *Z. mays*. The neighbor-joining (NJ) tree was constructed using Molecular Evolutionary Genetics Analysis Version 7.0 (MEGA7). The values shown at the branch nodes are the confidence levels from 1000 replicate bootstrap samplings. Red dots indicate the FRKs from *M. esculenta*, blue triangles indicate the FRKs from *J. curcas*, and green squares indicate the FRKs from *R. communis*.

To determine the evolutionary relationships among the plant FRK proteins, sequences of 38 FRK family members from *M. esculenta*, *Jatropha curcas*, *Ricinus communis*, *S. lycopersicum*, *A. thaliana*, *Camellia Sinensis*, *Saccharum spontaneum*, and *Z. mays* were analyzed using a neighbor-joining (NJ) phylogenetic tree (Figure 4). These plant FRK proteins were classified into two groups (α and β groups). MeFRK1, MeFRK2, MeFRK6, and MeFRK7 were clustered in the α group, wherein MeFRK1 and MeFRK7, MeFRK2 with JcFRK7, and MeFRK6 with RcFRK6 were clustered together in a subclade. MeFRK3–5 were clustered in the β group, wherein MeFRK3 and MeFRK4 had a close relationship with JcFRK2, and RcFRK4; MeFRK6 and RcFRK6 are clustered together in a subclade. Most MeFRK proteins have a close relationship with the FRK proteins from *J. curcas* and *R. communis*. The three species belong to *Euphorbiaceae*.

### 2.4. Motif Distribution in MeFRK Proteins

**Figure 5:**
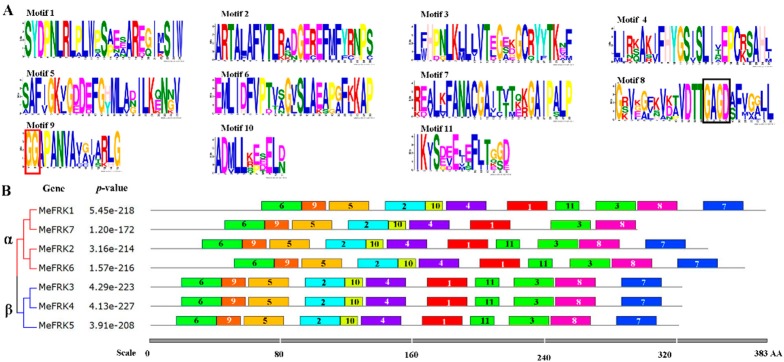
The conserved motifs of MeFRK proteins according to the phylogenetic relationships. (**A**) The motif sequences in MeFRKs, which were identified by MEME. The GG and G/AXGD motifs are indicated by red and black boxes, respectively; (**B**) the motif distribution in MeFRKs. The NJ tree was constructed with full amino acids of MeFRKs using Muscle and MEGA7 software with 1000 bootstraps. Gray lines represent the non-conserved sequences, and each motif is indicated by a colored box and a number. The length of the motifs in each protein is shown proportionally. α and β indicate different groups of MeFRKs.

To further examine the structural features of MeFRKs, the conserved motifs were analyzed according to their phylogenetic relationships. Eleven motif sequences have been identified in MeFRKs (Figure 5). The GG motif is found in Motif 9, and the G/AXGD motif is found in Motif 8 (Figure 5A). All the motifs distributed in the α or β MeFRK groups are similar, but the non-conserved sequences at the N-terminus are longer in the α group compared to the β group. MeFRK7 lost Motif 7 and Motif 11. Interestingly, MeFRK3 and MeFRK4 had the same motif distribution.

### 2.5. Three-Dimensional Structure of MeFRK Proteins

**Figure 6:**
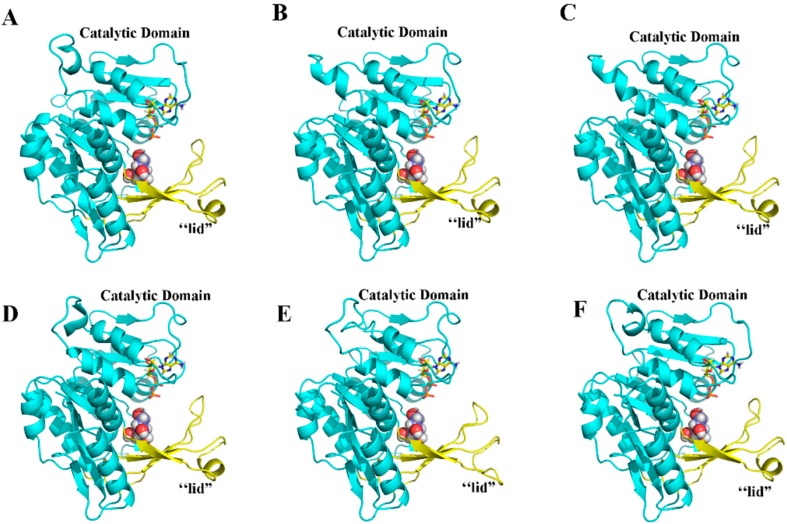
Illustrated representation and substrate recognition domains of the predicted three-dimensional structure models of MeFRK1–6. (**A**) MeFRK1, (**B**) MeFRK2, (**C**) MeFRK3, (**D**) MeFRK4, (**E**) MeFRK5, and (**F**) MeFRK6. The catalytic domains of the MeFRKs are depicted in blue, and the β-sheet ‘‘lid” regions in yellow. Colored stick structures represent ATP, and spherical structures indicate fructose molecules. The image was generated using the PyMOL program (Schrödinger, Inc., New York, NY, USA).

**Figure 7:**
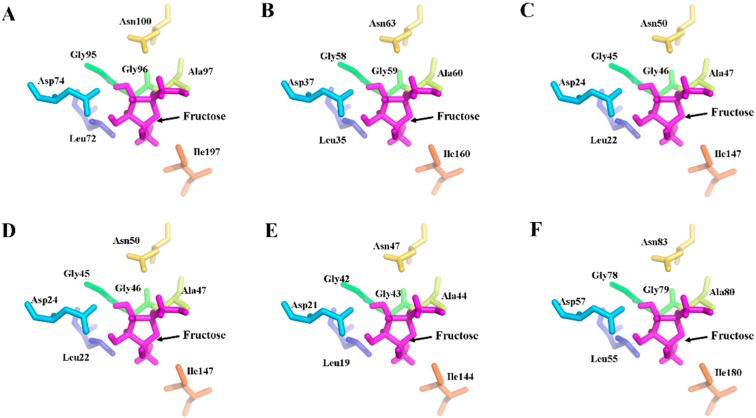
Distribution of the substrate binding residues of MeFRK1–6. (**A**) MeFRK1, (**B**) MeFRK2, (**C**) MeFRK3, (**D**) MeFRK4, (**E**) MeFRK5, and (**F**) MeFRK6. Purple stick structures indicate fructose molecules. The colored stick structures represent the substrate binding residues: leucine (Leu) indicated by blue, lspartic acid (Asp) indicated by light blue; glycine (gly) indicated by green, alanine (Ala) indicated by chartreuse, asparagine (Asn) indicated by yellow, isoleucine (Ile), indicated by brown. The image was generated using the PyMOL program (Schrödinger, Inc., New York, NY, USA).

To further characterize the MeFRKs, the three-dimensional (3D) structures of MeFRK1–6 proteins were modeled based on the crystal structure of FRK protein from *Halothermothrix orenii* (Protein Databank ID 3HJ6) using SWISS-MODEL software. The predicted 3D structures of MeFRK1–6 were validated with the QMEAN server for model quality estimation. The total QMEAN-score (estimated model reliability between 0 and 1) of the predicted 3D models for MeFRK1–6 were 0.49, 0.56, 0.56, 0.56, 0.57, and 0.50, respectively. This indicates that the predicted 3D structures of MeFRK1–6 were reliable. The overall three-dimensional structures of the six MeFRK proteins were similar (Figure 6). The sequences of the MeFRK proteins distinctly fold into the catalytic domains and a β-sheet ‘‘lid” region. To predict the theoretical position of the sites for fructose and ATP binding with MeFRK1–6, the primary models of MeFRK1–6 were further structurally aligned with a model of the FRK protein from *Vibrio cholerae* O395 (Protein databank ID 5EYN) using the PyMOL program (Schrödinger, New York, NY, USA). The results showed that ATP is predicted to bind a pocket on the catalytic domain, and the substrate binding cleft of MeFRK1–6 is located at the interface between the catalytic domain and the lid region (Figure 6). MeFRK1–6 contain all seven of the conserved substrate binding residues (using the numbering based on MeFRK1, the residues are Leu72, Asp74, Gly95, Gly96, Gly97, Asn100, and Ile197) and have a orientation similar to that of the fructose molecule (Figure 7). The numbers of substrate binding residues for MeFRK3 and MeFRK4 are the same (Figure 7C,D).

### 2.6. The Expression Patterns of MeFRK Genes in Different Tissues of Cassava

**Figure 8:**
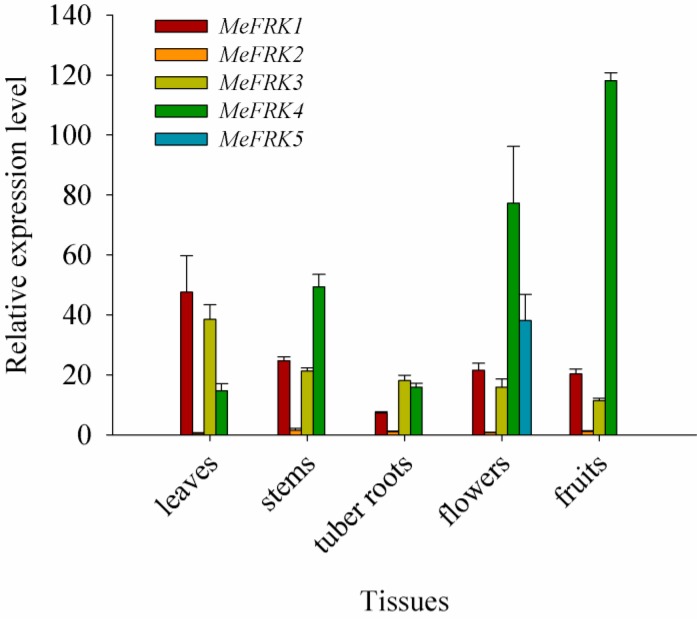
The differential expression of the *MeFRK* genes in cassava organs and tissues. The amount of *MeFRK* mRNA was normalized by *β-tubulin* mRNA. The expression level of *MeFRK2* in tuber roots, 180 days after planting, was used to calibrate the data for other genes for map-making. Each value is the mean ± standard error (SE) of three biological replicates (*n* = 3).

The tissue expression profiles of *MeFRK1-6* were determined in the leaves, stems, tuber roots, flowers, and fruits of cassava. All the tested tissues were collected 180 days after planting, except the fruit, which was collected 225 days after planting. The results show that the expressions of *MeFRK1*–*4* were identified in all tested tissues, whereas the expression level of *MeFRK2* in the tissues was comparably low. As for specific tissues and organs, the expression levels of *MeFRK1* and *MeFRK3* were high in the leaves, and that of *MeFRK4* was high in the reproductive organs (flowers and fruits); *MeFRK5* was specifically expressed in flowers, and *MeFRK6* was specifically and weakly expressed in leaves (data not shown). Remarkably, almost all the *MeFRKs* had low expression levels in tuber roots 180 days after planting (Figure 8).

### 2.7. The Differential Expression of MeFRKs during Cassava Tuber Root Development

**Figure 9:**
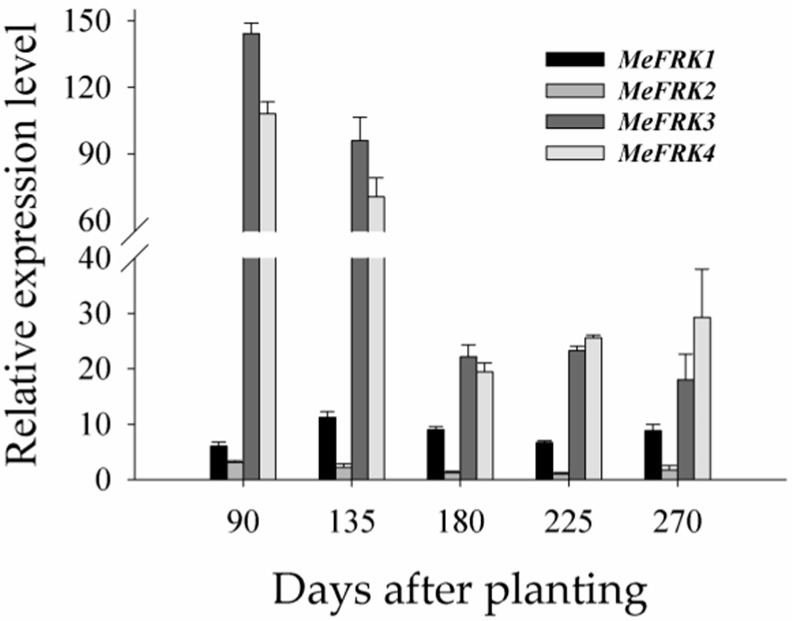
The differential expression analysis of *MeFRK* genes during cassava tuber root development. The differential expressions of *MeFRK* genes were examined using qPCR at the tuber initial stage (90 days), the tuber expanding stage (135 and 180 days), and the tuber maturity stage (225 and 270 days). Each value is the mean ± SE of three biological replicates (*n* = 3). The amount of *MeFRK* mRNA was normalized by *β-tubulin* mRNA. The expression level of *MeFRK2* at 180 days was used as a calibrator to compare the data across genes for map-making.

To investigate the expression patterns of *MeFRK* genes during tuber root developmental stages, the differential expressions of the *MeFRKs* were examined in tuber root 90, 135, 180, 225, and 270 days after planting. The results show that *MeFRK3* and *MeFRK4* were highly expressed at all stages, among which the highest expression was at the tuber initial stage (90 days), followed by the early expanding stage (135 days), and the expressions drastically reduced (almost a 4-fold decrease) at the later expanding stage (180 days) and tuber mature stages (225 and 270 days). The expression level of *MeFRK1* was more or less stable at all stages, but it was lower compared with *MeFRK3* and *MeFRK4*. A very weak expression of *MeFRK2* and no expression of *MeFRK5* or *MeFRK6* were observed in all cassava tuber development stages (Figure 9).

### 2.8. The Activity of FRKs in Cassava Tubers during Tuber Root Development

**Figure 10:**
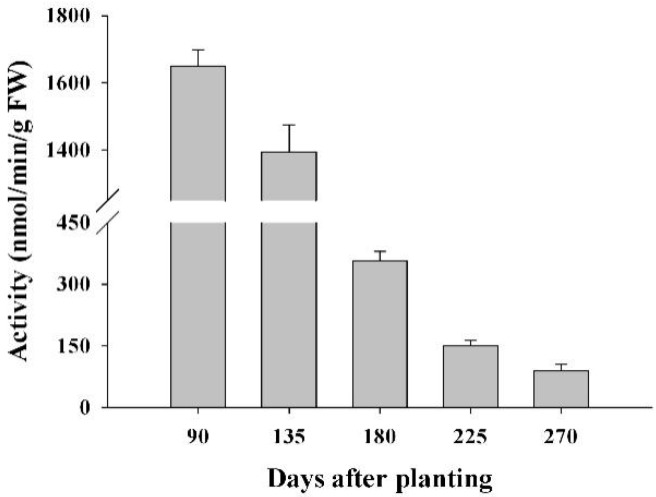
FRK activity profiles in cassava tubers during tuber root development at the initial tuber stage (90 days), the tuber expanding stage (135 and 180 days), and the tuber maturity stage (225 and 270 days). Each value is the mean ± standard error of three biological replicates (*n* = 3).

To test the possible involvement of FRKs in sucrose metabolism at a sink organ during cassava tuber root development, the FRK activity was measured at the initial tuber stage (90 days), the expanding tuber stage (135 and 180 days), and the mature tuber stage (225 and 270 days). The results showed that FRK activity in the tuber was highest at the initial tuber stage (90 days), followed by the early expanding tuber stage (135 days). It decreased with increasing tuber root maturity; thus, the lowest activity was found at the tuber mature stage, 270 days after planting (Figure 10).

### 2.9. Yeast Complementation of MeFRK3 and MeFRK4

**Figure 11:**
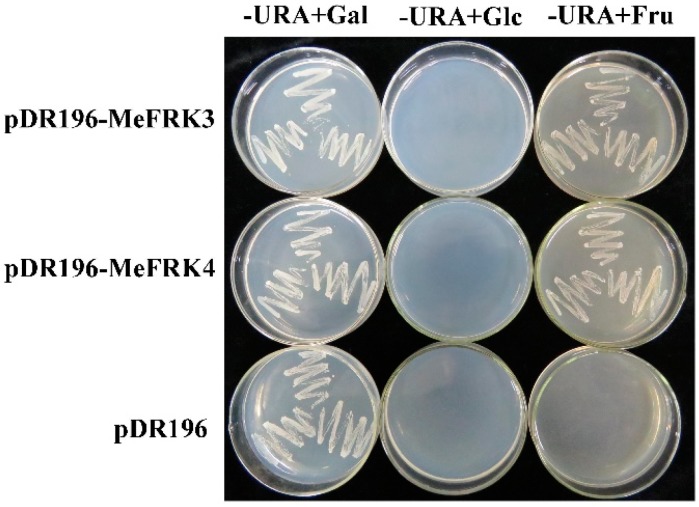
Complementation of the HXK-deficient yeast triple mutant YSH7.4-3C (*hxk1*, *hxk2*, *glk1*) with *MeFRK3* and *MeFRK4*. -URA + Gal: the medium contained galactose as the sole carbon source and lacked uracil; -URA + Glc: the medium contained d-glucose as the sole carbon source and lacked uracil; -URA + Fru: the medium contained d-fructose as the sole carbon source and lacked uracil. The yeast cells transformed with the vector alone (pDR196) were used as control.

To confirm the identity of MeFRKs as an FRK, the cDNAs of *MeFRK3* and *MeFRK4* were cloned into the yeast shuttle vector pDR196 to obtain pDR196-MeFRK3 and pDR196-MeFRK4. They were then expressed in a triple-mutant yeast strain YSH7.4-3C, which is a mutant for hexose kinases (*hxk1*, *hxk2*, *glk1*), unable to phosphorylate glucose or fructose. Therefore, YSH7.4-3C is unable to grow on the media containing fructose or glucose as the sole carbon sources. As shown in Figure 11, the yeast cells carrying pDR196-MeFRK3 and pDR196-MeFRK4 grew well on the medium with fructose, but could not grow on the medium with glucose; the control cells transformed with empty pDR196 vector failed to grow on either of the selection media. These results indicate that *MeFRK3* and *MeFRK4* encode FRKs with a high specificity to phosphorylate fructose.

## 3. Discussion

### 3.1. Identification and Characterization of MeFRK Genes

Fructokinase plays a key role in the tuberization of potato [22]. In addition, it has been reported that FRK is involved in the carbohydrate metabolism process during the tuberization of cassava roots [29,30]. However, no further information is available about the *FRK* gene family in cassava. Previous studies have reported that the members of the *FRK* gene family vary among plant species. For instance, it has seven members in *A. thaliana* [11], four members in *S. lycopersicum* [31], and two members in *Z. mays* [16]. In the present study, seven *FRK* genes (*MeFRK1–7*) were found in cassava, and six *MeFRKs* (*MeFRK1–6*) were cloned from cassava cultivar SC8. All the MeFRK proteins have the conserved domains of FRK (pfkB1 and pfkB2), the di-gly (GG) motif, the GAGD motif, and the proposed key substrate binding residues, which are consistent with the reported FRKs in other plants [11]. Phylogenetic analysis of the 38 FRK proteins from eight plants showed that the MeFRKs are most closely related to the FRKs from *R. communis* and *P. trichocarpa* (Figure 4). These three species belong to the order Malpighiales. The α group members (MeFRK1, –2, –6, and –7) have more exons and longer amino acid sequence than the β group members (MeFRK3–5) (Table 1, Figure 2). Simultaneously, motif analysis showed that the MeFRKs in the α group have longer non-conserved sequences at the N-terminus compared to the β group MeFRKs (Figure 5). These structural differences between α and β group members of MeFRKs might cause differences in their enzymatic activity. For example, the Km and kcat values of α group members (AtFRK1 and AtFRK3) in *A. thaliana* are higher compared to those of the β group members (AtFRK2–7) [11]. The 3D structural models of MeFRK1–6 showed that these proteins are distinctly folded into a catalytic domain and a β-sheet ‘‘lid” region. They form the substrate binding cleft that contains many residues involved in fructose binding, and ATP is predicted to bind a pocket on the catalytic domain. These structures are typical of FRK proteins [11,32]. Seven substrate binding residues of MeFRKs are conserved and have similar orientation with the fructose molecule. The results of the sequence and 3D structure analyses of MeFRKs suggest that all FRK members from cassava can catalyze the phosphorylation of fructose. 

### 3.2. Differential Expression and Enzymatic Activity of MeFRKs

The tissue-specific expression patterns of *MeFRK1–6* could provide a basis for understanding their functions in cassava plant development. The expression patterns of *MeFRK1–6* in various organs or tissues were examined at the tuber developmental stage (180 days). *MeFRK1–4* were widely expressed in most plant tissues. *MeFRK1* and *MeFRK3* were highly expressed in the leaves, suggesting that these two genes might play an essential role in leaf development. *MeFRK2* was expressed at low levels in all tested tissues, and *MeFRK6* was weakly expressed in leaves, which indicates that these two genes might not play an important role in the carbohydrate metabolism of the cassava plant. *MeFRK4* was highly expressed in the stems, flowers, and fruits, indicating that it might primarily regulate the development of stems, flowers, and fruits. Suppression of *SlFRK3* in tomato can reduce the stem xylem area, indicating that Slfrk3 may play a role in xylem development [13]. *MeFRK5* was specifically expressed in flowers, suggesting that this gene might have a specific function in floral development. This result is correlated with tomato *FRK4*, which is a pollen-specific expression [33]. The expressions of *MeFRKs* suggest that the roles of *MeFRKs* in different tissues might vary. Similar results have been found for other *FRK* genes from *S. lycopersicum* participating in the development of flowers, pollen, stems, leaves, root, and fruits [9].

The expression of *MeFRK7* was not detected in any tissues. It has been reported that the expression of some plant *FRK* genes are affected by abiotic stress, such as anaerobic stress [20] and salt stress [21]. Recently, the expression of *FRKs* in *C. sinensis* was found to respond to salt stress, drought stress, and cold stress [34]. In *Saccharum*, *FRK3* and *FRK5* were both dramatically induced under drought stress [35]. Therefore, we speculate that the expression of *MeFRK7* might be related to certain stressed conditions.

Our results show that almost all the *MeFRK* genes were weakly expressed in tuber roots, compared to other tissues, 180 days after planting. However, it has been reported that FRKs play a role in cassava tuber root development and starch synthesis [36]. FRKs were reportedly involved in the development of sink organs. In *Z. mays*, *ZmFRK2* is expressed at an earlier time during seed development [16]. In rice, *OsFKI* and *OsFKII* are especially expressed in rice grains and play a role in starch storage during development of rice grains [14]. To investigate the temporal expression of *MeFRK1–6* during cassava tuber root tuberization, qPCR analysis was performed using tubers at the initial tuber stage (90 days), the expanding tuber stage (135 and 180 days, the main period of starch accumulation), and the mature tuber stage (225 and 270 days). The results show that *MeFRK3* and *MeFRK4* were the most active genes among the *MeFRKs* in cassava tuber roots, with the highest expression level at the initial (90 days) and early expanding tuber stages (135 days). *MeFRK1* and *MeFRK2* maintained low expression levels, and *MeFRK5* and *MeFRK6* were not expressed in the tuber roots (Figure 9). These results show that *MeFRK3* and *MeFRK4* are the most active genes among *MeFRK1–6* in cassava tuber roots, and high expression is correlated with high fructokinase activity at the initial and early expanding tuber stages and lower activity at the mature tuber stages. Similarly, the expression of the *StFK1* gene in potato was high in small tubers, and declined after longer growth periods. Enzyme activity analysis found that the trend of fructose kinase activity during potato tuber development was consistent with the expression pattern of the *StFK1* gene [37]. Our results show that the enzymatic activity of FRKs was higher at the initial and expanding tuber stages in tuber roots, which is consistent with the results of *FRK* gene expression during cassava tuber root development (Figure 10). Potato FRK StFK1 mainly catalyzes the phosphorylation of fructose in tubers and participates in the tuberization and starch accumulation in tubers [22]. Modulation of the fructokinase activity of potato via antisense inhibition of the fructokinase resulted in a reduced tuber number and a reduced tuber yield [22]. The results from the gene expression and enzymatic activity analyses suggest that plastidic fructokinases *MeFRK3* and *MeFRK4* might be key genes for fructose phosphorylation during cassava tuber root development, and are involved in sucrose metabolism by regulating the formation of tuber roots and starch accumulation. Subsequent research will manipulate MeFRK activity in cassava to increase the number of tubers or the yield.

## 4. Materials and Methods

### 4.1. Plant Materials

Plants of cassava cultivar SC8 were used throughout the experiments described in this manuscript. The plants were field-grown at Hainan University under normal conditions at an average temperature of 23.8 °C and an average relative humidity of 85%. Leaves, stems, tubers, and flowers 180 days after planting, and fruits 225 days after planting, were collected for *MeFRK* gene cloning and for the investigation of their expression patterns in different cassava tissues. In order to identify the *FRK* members that play a key role in tuber root development, tuber tissues were collected for RNA extraction or FRK enzymatic analysis at the tuber root initial stage (90 days), the expanding stage (135, 180 days), and the maturity stage (225, 270 days). All samples were immediately frozen in liquid nitrogen upon collection and stored at −80 °C.

### 4.2. RNA Extraction and cDNA Synthesis

The total RNA was extracted using RNAplant Plus reagent (TianGen, Beijing, China) following the manufacturer’s instructions; it was electrophoresed in 1% agarose gel and stained with Golden View™ (BioMed, Beijing, China) to verify its quality. For gene clones, the reaction of reverse transcription was performed following the instructions on the RNA PCR Kit (AMV) Ver.3.0 and Oligo dT-Adaptor Primer (TaKaRa, Dalian, China). For quantitative real-time PCR analysis, reverse transcription was carried out with the PrimeScript™ RT Reagent Kit with gDNA Eraser (Perfect Real Time) (TaKaRa, Dalian, China), according to the manufacturer’s protocol.

### 4.3. Cloning of MeFRK cDNAs

Full-length cDNAs of the *MeFRK* genes were isolated via RT-PCR, using a set of gene-specific primers (Table 2), which were designed based on BLAST analysis of the cassava genome database (Available online: http://www.phytozome.net/cassava) using seven published sequences of the *AtFRK1–7* in *A. thaliana* [11]. The PCR reaction was carried out at a final volume of 50 μL, containing 1 μL of cDNA from different tissues, following the manufacturer’s instructions from the Ex Taq DNA polymerase kit (Takara, Japan). The PCR cycling conditions were as follows: 3 min at 94 °C, followed by 30 cycles of 94 °C for 30 s, a range of annealing temperatures for different *MeFRKs* from 57 to 63 °C for 30 s, 72 °C for 2 min, and a final extension of 10 min at 72 °C. The PCR products were separated on 1% agarose gel and purified by Axygen Purification kit (Axygen, Union, CA, USA), cloned into pMD18-T vector (Takara, Dalian, China), and sequenced (Shanghai Sangon Biological Engineering Technology and Services Co., Ltd, Shanghai, China).

### 4.4. Sequence Feature Analyses and Phylogeny Construction

The molecular weight (Mw) and theoretical isoelectric point (pI) of MeFRKs were predicted using ExPASy [38]. The multiple sequence alignment of the FRKs from cassava was carried out using the DNAman 6.0 program (Lynnon Biosoft, Quebec City, QC, Canada). The subcellular localizations of the MeFRKs were predicted by integrating the predictions by TargetP 1.1 (Available online: http://www.cbs.dtu.dk/services/TargetP/) and WoLF PSORT (Available online: http://www.genscript.com/wolf-psort.html). For the phylogenetic analysis, FRKs from *M. esculenta*, *J. curcas*, *R. communis, S. lycopersicum*, *A. thaliana*, *C. Sinensis*, *S. spontaneum*, and *Z. mays* were aligned using the Muscle program, and the phylogenetic tree was constructed using the MEGA 7 program [39]. The branching reliability was assessed by the bootstrap re-sampling method using 1000 bootstrap replicates. The conserved motifs of FRK proteins from *M. esculenta* were predicted using the MEME web server [40].

### 4.5. Exon–Intron Structure Analysis and Chromosomal Mapping 

In order to display the structure of introns and exons of *MeFRK* genes, the cDNA sequences of MeFRK genes were aligned with the corresponding genomic DNA sequences from the cassava genome database (Available online: http://www.phytozome.net/cassava). The Gene Structure Display Server (GSDS) program was used to visualize the gene structure [41]. The genomic position of the *MeFRK* genes and the total length of each chromosome were obtained from the cassava genome database. Subsequently, the *MeFRK* genes were manually mapped onto chromosomes.

### 4.6. Prediction of Three-Dimensional Structure of the MeFRK Proteins

Full-length amino acid sequences of the six cassava FRK proteins were submitted to the Swiss-Model server (Available online: http://beta.swissmodel.expasy.org/) to predict their three-dimensional structure. All the resulting models were based on their homology to the three-dimensional structure of the FRK protein from *Halothermothrix orenii* (Protein Databank ID 3HJ6). To predict the theoretical site of fructose binding with MeFRKs, the 3D structural models of MeFRKs were further structurally aligned with an FRK from *Vibrio cholerae* O395 (Protein databank ID 5EYN) in complex with fructose, using PyMOL (Schrödinger, Inc., New York, NY, USA). The predicted substrate binding residues of MeFRKs were also displayed using PyMOL.

### 4.7. Quantitative Real-Time PCR (qPCR) Analyses

qRT-PCRs were performed on an Applied Biosystems HT7900 apparatus (Applied Biosystems CA, USA), using the Takara SYBR® Premix ExTaq II (Tli RNaseH Plus) kit (Takara, Dalian, Japan) in a 384-well plate. Each reaction was run in a 10 μL volume, containing 5 μL of 2× SYBR ^®^ Premix Ex Taq II (Tli RNaseH Plus), 0.2 μL of ROX Reference Dye (50×), 0.2 μL of forward and reverse primers (10 μM), 0.4 μL of H_2_O, and 4 μL of template cDNA. All PCR reactions were performed at the following standard conditions: 1 min at 95 °C for one cycle, followed by 45 cycles of 95 °C for 5 s and 60 °C for 30 s. The dissociation curve was used to assess the amplification specificity. The results were analyzed using SDS2.4 software. The specific primers for each gene are shown in Table 3. Three technical replicates for each biological sample were analyzed. Tubulin was used as a reference gene, as described previously [42]. Relative abundance of the transcripts was analyzed using the 2^−ΔΔ*C*t^ method [43].

### 4.8. Activity Analysis of the Fructokinases

The FRK activity in tuber during cassava tuber developmental stages were measured using a continuous assay, coupling fructose phosphorylation to NADP^+^ reduction at 340 nm, according to Qin et al. [15]. Fructokinase activity was measured as the total fructose phosphorylating capacity and expressed in nmol/min/g FW. Three technical replicates for each biological sample were analyzed.

### 4.9. Yeast Complementation Assay for MeFRK3 and MeFRK4

A yeast shuttle vector pDR196, containing the URA3 gene as a selective marker, was used to express the *MeFRK3* and *MeFRK4* cDNAs in yeast cells. The cDNAs were inserted as a PstI/SalI fragment into the PstI/SalI sites within pDR196, and the resultant plasmids of pDR196-MeFRK3 and pDR196-MeFRK4 were verified by sequencing. Yeast (*Saccharomyces cerevisiae*) transformation was carried out by the LiAc/PEG method using the triple mutant yeast cells YSH7.4-3C (*hxk1*, *hxk2*, *glk1*) that are unable to phosphorylate either glucose or fructose [44]. The yeast cells were grown on YPgal medium, consisting of 2% bacto-peptone, 1% yeast extract, and 2% galactose. Selective media for uracil auxotrophic growth of the transformed colonies contained 0.67% yeast Nitrogen base (Difco) and 2% of either galactose, glucose, or fructose, supplemented with the appropriate amino acids and lacking uracil. The mutant strain transformed with an empty pDR196 vector served as the control. 

## Figures and Tables

**Table 1:** Characteristics of the cassava fructokinase proteins that were deduced from cDNA sequences.

Gene	Gene ID	Accession Numbers	Subcellular Localization	Size		pI	% Similarity (a.a.) to					
				a.a	kDa		MeFRK2	MeFRK3	MeFRK4	MeFRK5	MeFRK6	MeFRK7
*MeFRK1*	Manes.01G116600	AJK93565	Plastid	383	41.2	5.32	63.38	56.66	56.66	52.74	63.05	68.57
*MeFRK2*	Manes.06G141400	AJK93566	Cytoplasm	347	37.2	5.70	—	62.53	63.40	59.37	67.57	52.34
*MeFRK3*	Manes.11G121800	AJK93567	Plastid	331	35.3	4.99		—	93.05	66.57	58.38	50.14
*MeFRK4*	Manes.04G043700	AJK93568	Plastid	331	35.4	4.99			—	66.87	57.84	50.14
*MeFRK5*	Manes.15G189700	KR338981	Cytoplasm	329	35.0	5.73				—	56.76	46.69
*MeFRK6*	Manes.01G263700	AJK93569	Cytoplasm	370	40.1	6.07					—	­52.96
MeFRK7 *	Manes.02G075300	—	Plastid	303	32.9	6.62						—

* The characteristics of the MeFRK7 protein were analyzed according to predicate information from the cassava genome database.

**Table 2:** Gene specific primers of *MeFRKs* used for RT-PCR amplification.

Gene	Forward Primer (5′ to 3′)	Reverse Primer (5′ to 3′)
*MeFRK1*	TACCCATTAATTCTAACGCCAC	CATAAAACTAGGATTGCAGACATCT
*MeFRK2*	CTCTCTCATCTCTGATTCGTGCT	GGTTGTGAACTAAGAAGGATTGAA
*MeFRK3*	CTTCTTCTCTCATTCTCCTCTACAAA	TGCTACCCAAACAAAAAGAGAATAT
*MeFRK4*	GACTTCATTGTCTCTTCTTCTCTTCC	AATTTGTCTATTTTGAGGCGTTG
*MeFRK5*	AAAAAATGACAATCTCAGCAGC	AATTAGTTGGTGTTGGACTTGAT
*MeFRK6*	TTCTCATCTGATGTTGCCTGAC	CATTGTGAAATCTATCCTGCTCA
*MeFRK7*	TTCAACTGCATGGCTCTTCACTCTA	GAACAAGGAAATGGGAATACTGAAC

**Table 3:** Primers for *MeFRK1-6* used for qPCR amplification.

Gene	Forward Primer (5′ to 3′)	Reverse Primer (5′ to 3′)
*MeFRK1*	TTGCCTCCTCTTGTTTCCATTC	TTGCTGCATTTAACCCATCACC
*MeFRK2*	CAGGGTTGGTGGTGTGAAAGTG	ACCGCTCCTCATCCTTCAATAG
*MeFRK3*	CTTCTTCTCTCATTCTCCTCTACAA	TTCAAGAAACCAGGTGCCTC
*MeFRK4*	CCGAATCCTATTATCACGCG	GGAGACAGTGGGGACGAAGT
*MeFRK5*	CTCAGCAGCAAACAATAGCCCAT	TAGCACAGGCAACATTGGCAGGT
*MeFRK6*	GAATGTTTTCGATGCTGTTTATGTT	GCCAGGTGCTTCTGCAAGTG

## References

[1] Ruan Y.L. (2014). Sucrose metabolism: Gateway to diverse carbon use and sugar signaling. Annu. Rev. Plant Biol..

[2] Chen L.Q., Qu X.Q., Hou B.H., Sosso D., Osorio S., Fernie A.R., Frommer W.B. (2012). Sucrose efflux mediated by sweet proteins as a key step for phloem transport. Science.

[3] Winter H., Huber S.C. (2000). Regulation of sucrose metabolism in higher plants: Localization and regulation of activity of key enzymes. Crit. Rev. Biochem. Mol..

[4] Ni D.A. (2012). Role of vacuolar invertase in regulating arabidopsis stomatal opening. Acta Physiol. Plant.

[5] Fotopoulos V. (2005). Plant invertases: Structure, function and regulation of a diverse enzyme family. J. Biol. Res..

[6] Koch K. (2004). Sucrose metabolism: Regulatory mechanisms and pivotal roles in sugar sensing and plant development. Curr. Opin. Plant Biol..

[7] Halford N., Curtis T., Muttucumaru N., Postles J., Mottram D. (2011). Sugars in crop plants. Ann. Appl. Biol..

[8] Cho Y.H., Yoo S.D. (2011). Signaling role of fructose mediated by FINS1/FBP in *Arabidopsis thaliana*. PLoS Genet..

[9] Granot D., Kelly G., Stein O., David-Schwartz R. (2013). Substantial roles of hexokinase and fructokinase in the effects of sugars on plant physiology and development. J. Exp. Bot..

[10] Granot D., David-Schwartz R., Kelly G. (2013). Hexose kinases and their role in sugar-sensing and plant development. Front. Plant Sci..

[11] Riggs J.W., Cavales P.C., Chapiro S.M., Callis J. (2017). Identification and biochemical characterization of the fructokinase gene family in *Arabidopsis thaliana*. BMC Plant Biol..

[12] Pego J.V., Smeekens S. (2000). Plant fructokinases: A sweet family get-together. Trends Plant Sci..

[13] Stein O., Damari-Weissler H., Secchi F., Rachamilevitch S., German M.A., Yeselson Y., Amir R., Schaffer A., Holbrook N.M., Aloni R. (2016). The tomato plastidic fructokinase SlFRK3 plays a role in xylem development. New Phytol..

[14] Jiang H., Dian W., Liu F., Ping W. (2003). Isolation and characterization of two fructokinase cDNA clones from rice. Phytochemistry.

[15] Qin Q.P., Zhang J.W., Xie M., Jin Y.F., Chen K.S., Asghar S. (2004). Isolation and expression analysis of fructokinase genes from citrus. Acta Physiol. Plant.

[16] Zhang S., Nichols S.E., Dong J.G. (2003). Cloning and characterization of two fructokinases from maize. Plant Sci..

[17] Roach M., Gerber L., Sandquist D., Gorzsás A., Hedenström M., Kumar M., Steinhauser M.C., Feil R., Daniel G., Stitt M. (2012). Fructokinase is required for carbon partitioning to cellulose in aspen wood. Plant J..

[18] Odanaka S., Bennett A.B., Kanayama Y. (2002). Distinct physiological roles of fructokinase isozymes revealed by gene-specific suppression of *Frk1* and *Frk2* expression in tomato. Plant Physiol..

[19] David-Schwartz R., Weintraub L., Vidavski R., Zemach H., Murakhovsky L., Swartzberg D., Granot D. (2013). The SlFRK4 promoter is active only during late stages of pollen and anther development. Plant Sci..

[20] Guglielminetti L., Morita A., Yamaguchi J., Loreti E., Perata P., Alpi A. (2006). Differential expression of two fructokinases in *Oryza sativa* seedlings grown under aerobic and anaerobic conditions. J. Plant Res..

[21] Christian Z.R., Sigrid S., Mühling K.H. (2010). Proteomic changes in maize roots after short-term adjustment to saline growth conditions. Proteomics.

[22] Davies H.V., Shepherd L.V., Burrell M.M., Carrari F., Urbanczyk-Wochniak E., Leisse A., Hancock R.D., Taylor M., Viola R., Ross H. (2005). Modulation of fructokinase activity of potato (*Solanum tuberosum*) results in substantial shifts in tuber metabolism. Plant Cell Physiol..

[23] Comparot-Moss S., Denyer K. (2009). The evolution of the starch biosynthetic pathway in cereals and other grasses. J. Exp. Bot..

[24] Yang M., Dong J., Zhao W., Gao X. (2016). Characterization of proteins involved in early stage of wheat grain development by iTRAQ. J. Proteom..

[25] Agrawal L., Chakraborty S., Jaiswal D.K., Gupta S., Datta A., Chakraborty N. (2008). Comparative proteomics of tuber induction, development and maturation reveal the complexity of tuberization process in potato (*Solanum tuberosum* l.). J. Proteome Res..

[26] Santa Brígida A.B., dos Reis S.P., Costa C.d.N.M., Cardoso C.M.Y., Lima A.M., de Souza C.R.B. (2014). Molecular cloning and characterization of a cassava translationally controlled tumor protein gene potentially related to salt stress response. Mol. Biol. Rep..

[27] Oyelakin O.O., Opabode J.T., Raji A.A., Ingelbrecht I.L. (2015). A cassava vein mosaic virus promoter cassette induces high and stable gene expression in clonally propagated transgenic cassava (*Manihot esculenta* crantz). S. Afr. J. Bot..

[28] Ihemere U., Arias-Garzon D., Lawrence S., Sayre R. (2006). Genetic modification of cassava for enhanced starch production. Plant Biotechnol. J..

[29] Wang X., Chang L., Tong Z., Wang D., Yin Q., Wang D., Jin X., Yang Q., Wang L., Sun Y. (2016). Proteomics profiling reveals carbohydrate metabolic enzymes and 14-3-3 proteins play important roles for starch accumulation during cassava root tuberization. Sci. Rep..

[30] Sheffield J., Taylor N.C., Chen S. (2006). The cassava (*Manihot esculenta* crantz) root proteome: Protein identification and differential expression. Proteomics.

[31] Granot D. (2007). Role of tomato hexose kinases. Funct. Plant Biol..

[32] Chua T.K., Seetharaman J., Kasprzak J.M., Ng C., Patel B.K., Love C., Bujnicki J.M., Sivaraman J. (2010). Crystal structure of a fructokinase homolog from *Halothermothrix orenii*. J. Struct. Biol..

[33] German M.A., Dai N., Chmelnitsky I., Sobolev I., Salts Y., Barg R., Schaffer A.A., Granot D. (2002). LeFRK4, a novel tomato (*Lycopersicon esculentum* mill.) fructokinase specifically expressed in stamens. Plant Sci..

[34] Li N.N., Qian W.J., Wang L., Cao H.L., Hao X.Y., Yang Y.J., Wang X.C. (2017). Isolation and expression features of hexose kinase genes under various abiotic stresses in the tea plant (*Camellia sinensis*). J. Plant Physiol..

[35] Chen Y., Zhang Q., Hu W., Zhang X., Wang L., Hua X., Yu Q., Ming R., Zhang J. (2017). Evolution and expression of the fructokinase gene family in *saccharum*. BMC Genom..

[36] Zou M., Cheng L., Zhang S., Chen Q., Sun X., Ma P., Hu M., Peng M., Ma Z., Chen X. (2017). Epigenetic map and genetic map basis of complex traits in cassava population. Sci. Rep..

[37] Ross H.A., Davies H.V., Burch L.R., Viola R., Mcrae D. (1994). Developmental changes in carbohydrate content and sucrose degrading enzymes in tuberising stolons of potato (*Solanum tuberosum*). Physiol. Plant.

[38] Gasteiger E., Gattiker A., Hoogland C., Ivanyi I., Appel R.D., Bairoch A. (2003). Expasy: The proteomics server for in-depth protein knowledge and analysis. Nucleic Acids Res..

[39] Kumar S., Stecher G., Tamura K. (2016). Mega7: Molecular evolutionary genetics analysis version 7.0 for bigger datasets. Mol. Bio. Evol..

[40] Bailey T.L., Boden M., Buske F.A., Frith M., Grant C.E., Clementi L., Ren J., Li W.W., Noble W.S. (2009). Meme suite: Tools for motif discovery and searching. Nucleic Acids Res..

[41] Guo A.Y., Zhu Q.H., Chen X., Luo J.C. (2007). gsds: A gene structure display server. Hereditas.

[42] Yao Y., Geng M.T., Wu X.H., Liu J., Li R.M., Hu X.W., Guo J.C. (2015). Genome-wide identification, expression, and activity analysis of alkaline/neutral invertase gene family from cassava (*Manihot esculenta* crantz). Plant Mol. Biol. Rep..

[43] Livak K.J., Schmittgen T.D. (2001). Analysis of relative gene expression data using real-time quantitative PCR and the 2^−ΔΔ*C*t^ method. Methods.

[44] Winde J.H., Crauwels M., Hohmann S., Thevelein J.M., Winderickx J. (1996). Differential requirement of the yeast sugar kinases for sugar sensing in establishing the catabolite-repressed state. Eur. J. Biochem..

